# Wear-Resistance Improvement of 65Mn Low-Alloy Steel through Adjusting Grain Refinement by Cyclic Heat Treatment

**DOI:** 10.3390/ma14247636

**Published:** 2021-12-11

**Authors:** Ying Tong, Yu-Qing Zhang, Jiang Zhao, Guo-Zheng Quan, Wei Xiong

**Affiliations:** 1 School of Intelligent Manufacturing and Automobile, Chongqing College of Electronic Engineering, Chongqing 401331, China; tongying2004@126.com; 2 Chongqing Key Laboratory of Advanced Mold Intelligent Manufacturing, School of Material Science and Engineering, Chongqing University, Chongqing 400044, China; zhangyuqing620@sina.com (Y.-Q.Z.); 201709021061@cqu.edu.cn (J.Z.); 3 Nanjing Jiepin Intelligent Technology Co., Ltd., Nanjing 210028, China; 4 Key Laboratory of Advanced Reactor Engineering and Safety of Ministry of Education, Collaborative Innovation Center of Advanced Nuclear Energy Technology, Institute of Nuclear and New Energy Technology, Tsinghua University, Beijing 100084, China

**Keywords:** 65Mn low-alloy steel, cyclic heat treatment, grain refinement, microstructure evolution, hardness, wear resistance

## Abstract

Refined microstructures achieved by cyclic heat treatment significantly contribute to improving the wear resistance of steels. To acquire the refined microstructures of 65Mn low-alloy steel, first, the specimens were solid solution-treated; then, they were subjected to cyclic heat treatment at cyclic quenching temperatures of 790–870 °C and quenching times of 1–4 with a fixed holding time of 5 min. The mechanical properties of 65Mn low-alloy steel in terms of hardness, tensile strength, elongation and wear resistance were characterized. Afterwards, the effect of cyclic heat treatment on microstructure evolution and the relationships between grain refinement and mechanical properties’ improvement were discussed. The results show that the average grain size firstly decreased and then increased with the increase in the quenching temperature. Hardness increased with grain refinement when the temperature was lower than 830 °C. Once the temperature exceeded 830 °C, hardness increased with the temperature increase owing to the enrichment of carbon content in the martensite. With the increase in cyclic quenching times, hardness continuously increased with grain refinement strengthening. In addition, both tensile strength and elongation could be significantly improved through grain refinement. The relationships among wear loss, hardness and average grain size showed that wear resistance was affected by the synthesis reaction of grain refinement and hardness. Higher hardness and refined grain size contributed to improving the wear resistance of 65Mn low-alloy steel.

## 1. Introduction

A typical spring steel, 65Mn low-alloy steel possesses dual mechanical properties of high-carbon steel and low-alloy steel involving high strength and hardness, excellent elasticity and hardenability, and superior wear resistance [1,2,3]. In addition to its use in the making of spring products, 65Mn low-alloy steel is a specified material for rotary blades listed in the national standard GB/T 5669-2008 [4]. Meanwhile, it is also one of the desirable materials for manufacturing the friction clutches and brakes of large engineering machinery such as tracked armored vehicles, cranes and loaders [3,5,6]. Due to the long-term alternating stress and heavy-loaded working conditions, excessive wear is the primary failure mode in these components [3,7]. Hence, improving the comprehensive mechanical properties in terms of hardness and wear resistance is desirable for 65Mn low-alloy steel. Grain refinement, as an important strengthening mechanism, not only enhances the strength and hardness of steels, but also simultaneously improves their toughness and plasticity [8,9,10]. When grain size is refined to microns or even sub-microns, the comprehensive mechanical properties of steels are significantly boosted [11,12]. Therefore, preparing refined microstructures of 65Mn low-alloy steel is critical to improving the wear resistance and service performances of such components.

So far, the main methods for acquiring grain refinement microstructures include alloying element addition [13], thermo-mechanical-controlled processing (TMCP) technology [14], magnetic-field and electric-field processing [15,16], cyclic heat treatment [17,18,19], etc. Among them, cyclic heat treatment has been applied to many steels and alloys to refine grain size and further enhance their mechanical performance without thermo-mechanical treatment or adding alloying elements. This technique involves repeated heating and quenching processes. In each cycle, the steel is heated to a critical temperature and held at that temperature for a short duration, followed by quenching and cooling to room temperature. The crucial cyclic heat treatment parameters, including temperature, holding time, quenching rate and cycles, directly influence the final microstructure morphology and grain refinement effect, thereby determining the mechanical properties of steels. Atanu Saha et al. [20] characterized the microstructures and mechanical properties of 0.6 wt% carbon steel in cyclic heat treatment with different cycles, from one to eight. It was found that, after eight cycles of heat treatment, the microstructures with fine ferrite grains and spheroidized cementite exhibited an excellent combination of strength and ductility. In another work, by Atanu Saha et al. [21], on an annealed hypereutectoid 1.24 wt% carbon steel, the result shows that, with the increase in heat treatment cycles, more uniformly dispersed finer cementite particles in austenite matrix were obtained at 894 °C. Grange et al. [22] suggested that grain refinement subjected to cyclic heat treatment improved both the strength and toughness properties of martensitic stainless steel. Alok Mishra et al. [23] also reported that the considerable improvements of hardness and strength for AISI 1080 steel were achieved by employing cyclic heat treatment with repeated short duration at 775 °C, followed by oil quenching. Agnieszka Kościelna [24] investigated the effect of cycle heat treatment parameters on the grain refinement of the Ti–48Al–2Cr–2Nb alloy and refined microstructures with eight-fold reduction in average grain size were obtained after cyclic heat treatment with five cycles. These works also confirmed that cyclic heat treatment holds tremendous potential for obtaining superior comprehensive mechanical properties through grain refinement microstructures. Nevertheless, it is noteworthy that the effect of cyclic heat treatment parameters on grain refinement is extremely complicated; more importantly, the wear resistance of steels is not completely linearly correlated with grain size, but it is also determined by hardness and strength [25]. Therefore, it is necessary to explore the microstructure evolution with cyclic heat treatment parameters and further uncover the relationships among wear resistance, hardness and grain refinement. These will provide significant guidance for improving the wear resistance of 65Mn low-alloy steel through adjusting grain refinement by cyclic heat treatment.

In this work, a series of cyclic heat treatment experiments with varying quenching temperature and quenching times were designed and conducted to prepare refined microstructures of 65Mn low-alloy steel. Mechanical properties’ tests, including hardness, tensile and wear experiments of 65Mn low-alloy steel subjected to cyclic heat treatment, were performed. Subsequently, the effects of quenching temperature and quenching times on variations in average grain size and grain refinement degree were analyzed and the relationships among hardness, average grain size and cyclic heat treatment parameters were revealed. The relationships between grain refinement and the improvement of tensile strength and elongation were analyzed. The friction coefficient and wear loss were evaluated to detail the effects of refined microstructures on wear resistance. Finally, the relationships among wear loss, hardness and grain refinement were uncovered.

## 2. Principles of Cyclic Phase Transformation Heat Treatment

**Figure 1:**
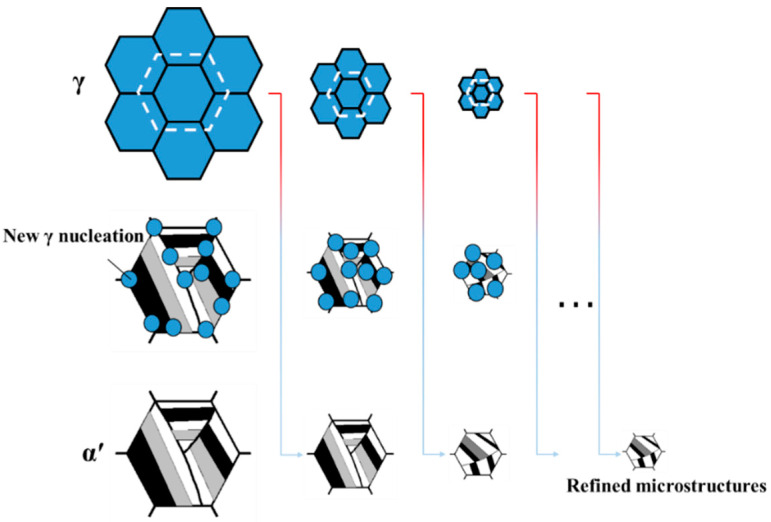
Principles of grain refinement in cyclic phase transformation heat treatment for 65Mn low-alloy steel.

During the cyclic heat treatment process of 65Mn low-alloy steel, austenite grains prefer to nucleate at higher stored energy regions, such as the original austenite grain boundary, dislocation and impurity in heating period, following the diffusionless phase transformation of austenite to martensite, which takes place in the quenching period [26]. It is generally known that martensite phase transformation is a shear transformation, during which the massive micro-defects of dislocation, stacking fault and twin generate [1]. Such micro-defects are further inherited in the reverted transformation of martensite to austenite in latter cycle heat treatment. Not only it can increase the stored energy of austenite and accelerate its nucleation in non-nucleated regions, but it can also increase the driving force of phase transformation and facilitate the next cyclic phase transformation. The repeated phase transformation of austenite to martensite causes austenite grain refining; thus, the refined microstructures are finally obtained. A schematic illustration of the grain refinement mechanism in cyclic phase transformation heat treatment for 65Mn low-alloy steel is shown in Figure 1.

**Figure 2:**
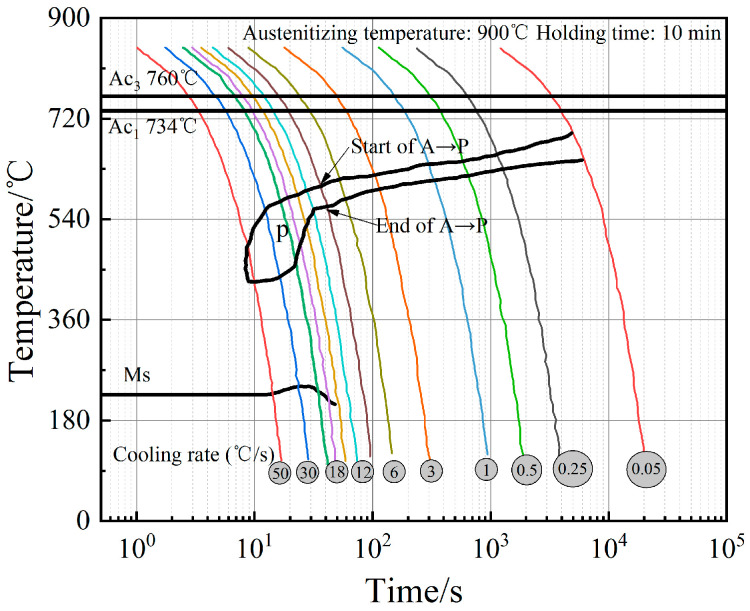
CCT curve of 65Mn low-alloy steel.

For a cyclic phase transformation heat treatment, the crucial heat treatment parameters, including heating rate, holding time, quenching temperature and quenching times, etc., have a strong influence on grain refinement. It is well accepted that the quenching temperature is determined by the critical temperature of Ac_1_ or Ac_3_. If the temperature lowers below Ac_1_, austenitization does not occur. On the contrary, austenite grains are coarser at a relatively higher temperature (above Ac_3_ would be too much) [26]. Generally, the quenching temperature is Ac_3_ + (30–50) °C for a hypo-eutectoid steel and Ac_3_ + (50–100) °C for a low-alloy steel. Since 65 Mn low-alloy steel is a hypo-eutectoid steel, its quenching temperature is determined as Ac_3_ + (30–100) °C. In order to obtain the complete phase transformation point of alloys, according to the volume expansion or shrinkage in phase transformation process, the CCT curve of 65Mn low-alloy steel was drawn using the thermal dilatation test and tangent methods, as shown in Figure 2. Then, the critical temperature of Ac_3_ was 760 °C and the critical cooling rate for acquiring martensite was 50 °C/s. With that, the optimal quenching temperature region for 65Mn low-alloy steel was identified as 790–860 °C in this work. Moreover, two vital parameters, holding time and heating rate, are also closely associated with refined austenite microstructures; further, a faster austenitizing rate and a lower holding time correspond to a grain growth that is as inconspicuous as possible. Usually, holding time *t* can be calculated by an experience formula such as Equation (1), where *α* is the heating coefficient, *K* is the furnace coefficient and *D* is the workpiece’s diameter.

(1) t=α·K·D

To ensure complete austenitization and reduce time, the holding time was identified as 5 min. The maximal heating rate was adopted and taken as 10 °C/s. In addition, it is widely known that, with the increase in cyclic quenching times, austenite grain size can be refined significantly, while the grain refinement effect becomes weaker and weaker. Here, the maximal cyclic quenching times for 65Mn low-alloy steel was set as four times.

## 3. Experiment Procedures

### 3.1. Experiment Schedule of Cyclic Heat Treatment

**Figure 3:**
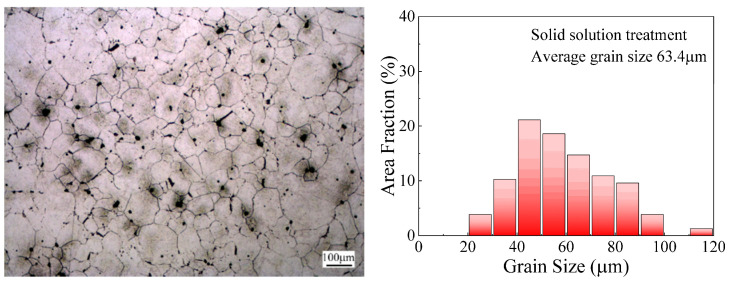
Microstructures and grain size distribution of 65Mn low-alloy steel subjected to solid solution treatment.

The studied material in this work is 65Mn low-alloy steel, whose chemical composition is given in Table 1. The as-received billet was a hot-rolled bar with a size of ø75 mm × 100 mm; its microstructures were composed of ferrite and pearlite. Ten cylindrical specimens 10 mm in diameter and 12 mm in height were machined from the billet by wire-electrode cutting. In order to make the specimens austenitize more completely and improve the distribution of composition in the alloy and uniformity of the microstructure, all specimens were solid solution-treated at the temperature of 900 °C for 60 min, followed by oil quenching. This process was performed to make preparations for the following cyclic heat treatment experiments. The solid solution-treated microstructure was characterized as shown in Figure 3, in which the austenite grains are distributed uniformly with an average grain size of 63.4 μm.

**Figure 4:**
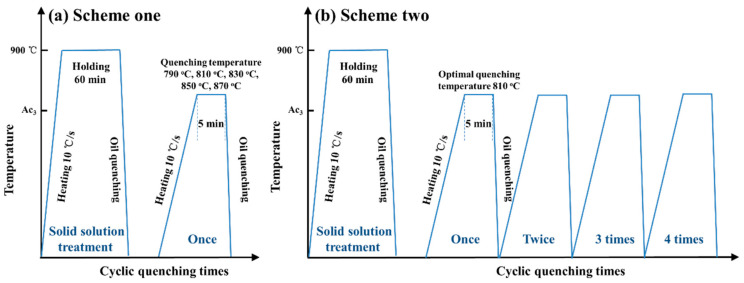
Processing routes for cyclic heat treatment of 65Mn low-alloy steel, (**a**) Scheme one; (**b**) Scheme two.

Based on the principles of cyclic heat treatment, an experiment scheme, with five different quenching temperatures of 790 °C, 810 °C, 830 °C, 850 °C and 870 °C, a fixed holding time of 5 min and a fixed cyclic quenching time of 1 time, was designed. Moreover, another experiment scheme, with four different cyclic quenching times of 1, 2, 3 and 4 times; a fixed optimal quenching temperature; and a fixed holding time of 5 min, was designed. In each cycle, the specimen was heated to a proposed quenching temperature with a heating rate of 10 °C/s and held at that temperature for 5 min in an electric resistance furnace, then immediately quenched in oil. The experimental procedures of cyclic phase transformation heat treatment are exhibited in Figure 4. The optimal cyclic quenching temperatures and times were determined by the analysis of grain morphology and austenite grain size.

After cyclic phase transformation heat treatment, the specimens were grinded and mechanically polished with successive grades of emery papers and diamond paste, followed by chemical corrosion in a solution consisting of saturated picric acid and sodium dodecyl benzene sulfonate detergent at the temperature of 60–70 °C for 80–100 s. Finally, the microstructures were observed using metallurgical optical microscope (OM). The statistical analysis of average grain size was conducted with the intercept method in image-pro plus software. Then, the grain refinement degree *δ* was calculated using Equation (2) to characterize the refinement effect of cyclic heat treatment under various cyclic quenching temperatures and times [27].

(2) $$\delta =\frac{d_{0}-d}{d_{0}}\times 100\%$$

where *δ* is the grain refinement degree; *d*_0_ is the initial average grain size and, in this work, *d*_0_ is the average austenite grain size after solid solution treatment, i.e., 63.4 μm; *d* is the average grain size after cyclic phase transformation heat treatment.

### 3.2. Characterization of Mechanical Performance

**Figure 5:**
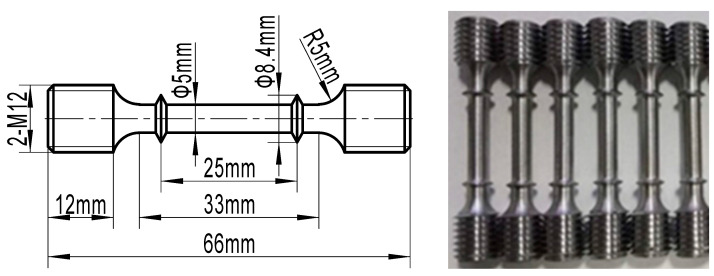
Standard cylindrical tensile specimens of 65Mn low-alloy steel for tensile tests.

The mechanical performance of the specimens after cyclic heat treatment was characterized. Hardness tests were performed on an HR150A Rockwell (Laizhou Lailuote Testing Instrument Co., Ltd., Yan tai, China) hardness tester equipped with a diamond cone-type indenter using a load of 1471 N. For each specimen, five points were tested and the average value was taken as the final hardness of the specimen. To characterize the tensile properties of 65Mn low-alloy steel subjected to various cyclic heat treatment conditions, tensile tests were conducted using a WDW-100 electronic universal testing machine. According to the Chinese national standard GB/T 4338-2006 [28], standard cylindrical tensile specimens processing various average grain sizes were machined to the dimensions shown in Figure 5. During the tensile test process, the specimens were stretched to break at the temperatures of 400 °C and 600 °C with a fixed strain rate of 1 s^−1^. The load and displacement data were monitored continuously by a computer control data acquisition system; then, the stress–strain curve could be generated from the recorded data.

**Figure 6:**
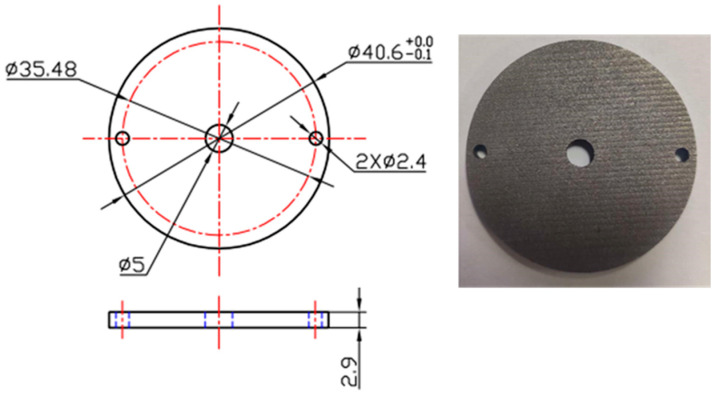
Standard specimens of 65Mn low-alloy steel for wear tests.

**Figure 7:**
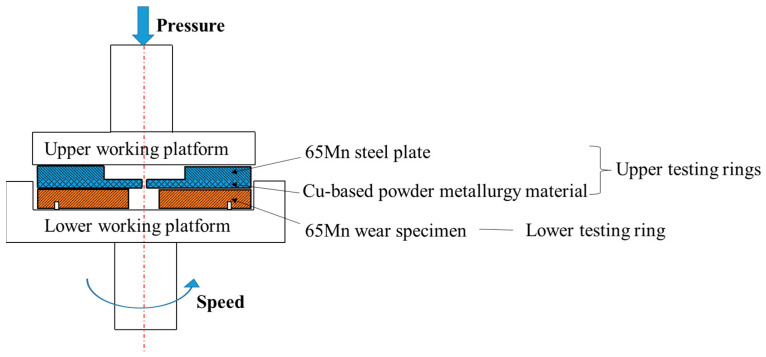
Schematic illustration of wear tests.

In order to characterize the effects of the refined microstructures on wear resistance, four specimens subjected to various cyclic heat treatment conditions were machined according to the geometry dimensions exhibited in Figure 6. Moreover, these specimens were prepared for wear tests carried on a UTM-5 (Beijing FHJR Scientific Technology Co., Ltd, Beijing, China) wear-testing machine. The procedures of the wear tests refer to the ASTM standard G99 [29]. A schematic illustration of the wear tests is shown in Figure 7. First, the wear specimen of 65Mn low-alloy steel (lower testing ring) was placed on the lower working platform and positioned by tow pins. The upper testing ring, including Cu-based powder metallurgy material and a 65Mn steel plate, was mounted on the upper working platform. The lower testing ring was driven by the working platform with a rotating speed of 850 r/min. Following that, the upper testing ring was pressed slowly with a load of 280 N until the upper and lower testing rings made contact completely. At this time, severe friction and wear behaviors occurred between the upper and lower testing rings and the pressure was held until the wear test finished. The friction coefficient and wear loss were characterized to evaluate the wear resistance of 65Mn low-alloy steel with different refined microstructures.

## 4. Microstructures of Specimens Subjected to Cyclic Heat Treatment

### 4.1. Effects of Cyclic Quenching Temperature on Microstructures

**Figure 8:**
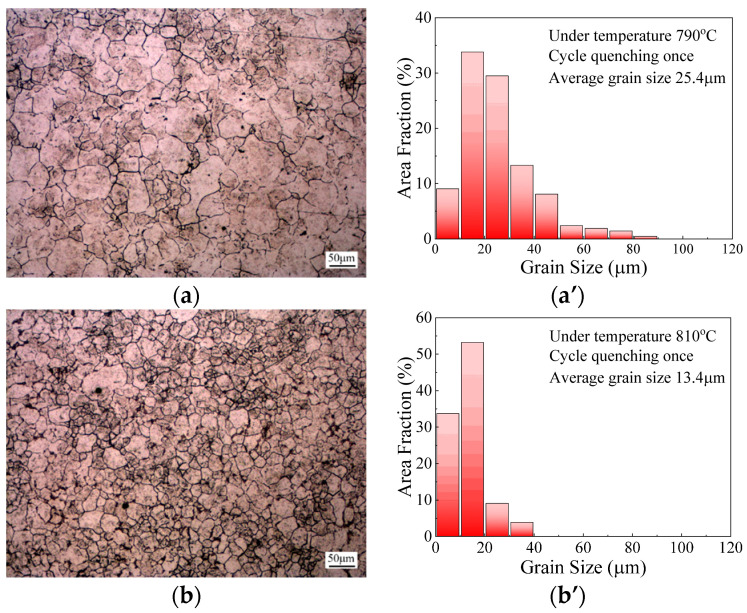

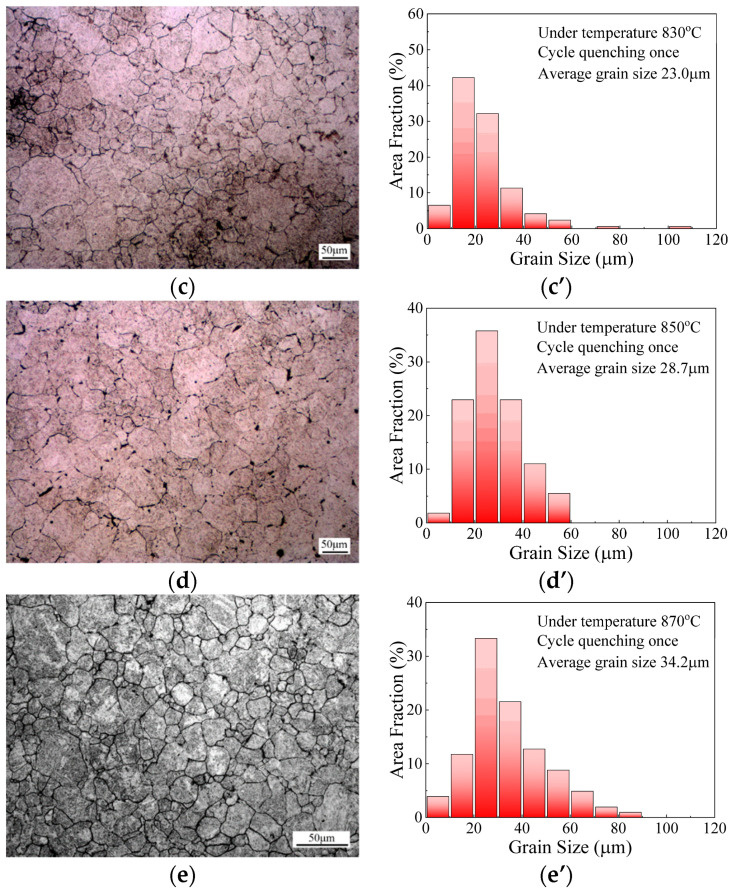
Microstructures and grain size distributions of specimens subjected to single quenching treatment under different cyclic quenching temperatures: (**a**,**a’**) 790 °C; (**b**,**b’**) 810 °C; (**c**,**c’**) 830 °C; (**d**,**d’**) 850 °C; (**e**,**e’**) 870 °C.

To analyze the effect of cyclic quenching temperature on microstructure evolution, the specimens were subjected to a single quenching treatment with a fixed holding time of 5 min under the four different quenching temperatures of 790 °C, 810 °C, 830 °C, 850 °C and 870 °C. Correspondingly, the microstructures and grain size distributions under these cyclic quenching conditions were obtained, as shown in Figure 8a–e. It can be observed, from Figure 8, that the austenite grains were refined significantly through cyclic heat treatment compared with those subjected to solid solution treatment. The average grain size at the cyclic quenching temperatures was measured as 25.4 μm, 13.4 μm, 23.0 μm, 28.7 μm and 34.2 μm and, correspondingly, the grain refinement degrees were calculated as 59.9%, 78.9%, 63.7%, 54.7% and 46.1%, respectively.

**Figure 9:**
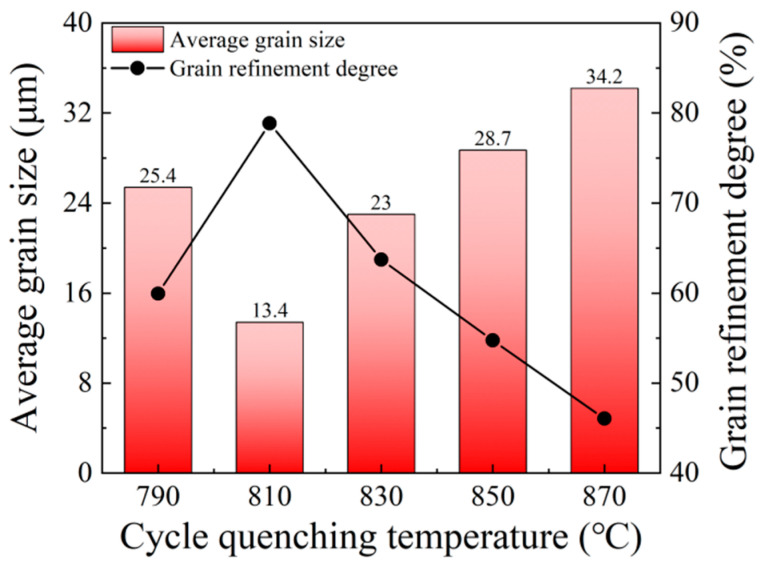
Variations in average grain size and grain refinement degree with cyclic quenching temperature.

Figure 9 exhibits the variations in average grain size and grain refinement degree with cyclic quenching temperatures. It can be seen that, with the increase in cyclic quenching temperature, the average grain size first decreased and then increased, while grain refinement demonstrated an opposite tendency. During the cyclic heat treatment of 65Mn low-alloy steel, the phase transformation between martensite and austenite occurred, with the generation of micro defects such as dislocation and twin, thus provoking the increase in stored energy. Afterwards, the recrystallized grains generated and then grew in the high stored-energy regions. As the quenching temperature increased from 790 °C to 810 °C, the grain refinement degree increased and grain size distribution became more homogeneous. This is attributable to the fact that, with the increase in quenching temperature, more potential nucleation sites were activated during the austenitization process. In addition, the driving force for the martensite-to-austenite transformation increased at a higher quenching temperature, which accelerated the recrystallization process and broke the structure inheritance. Hence, grain refinement was more sufficient with the increase in quenching temperature. However, once the cyclic quenching temperature exceeded 810 °C, due to the high grain boundary mobility provided by the elevated temperature, grain growth dominated and the newly formed recrystallized grains intended to grow in size. Therefore, grain size increased significantly; meanwhile, the grain refinement degree decreased with the further increase in the temperature. In conclusion, the microstructures of 65Mn low-alloy steel could be significantly refined and the grain refinement degree reached the maximum at 810 °C. Thus, the optimal cyclic quenching temperature was determined as 810 °C.

### 4.2. Effects of Cyclic Quenching Times on Microstructures

**Figure 10:**
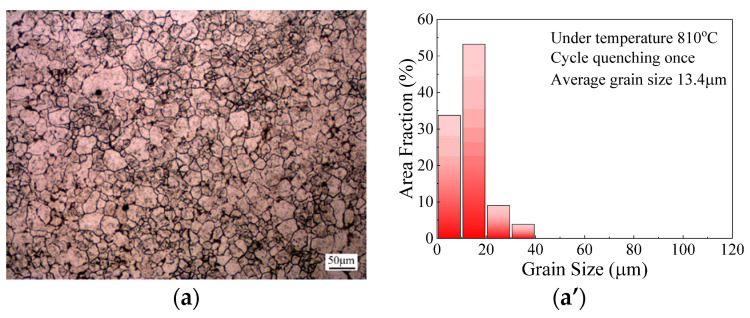

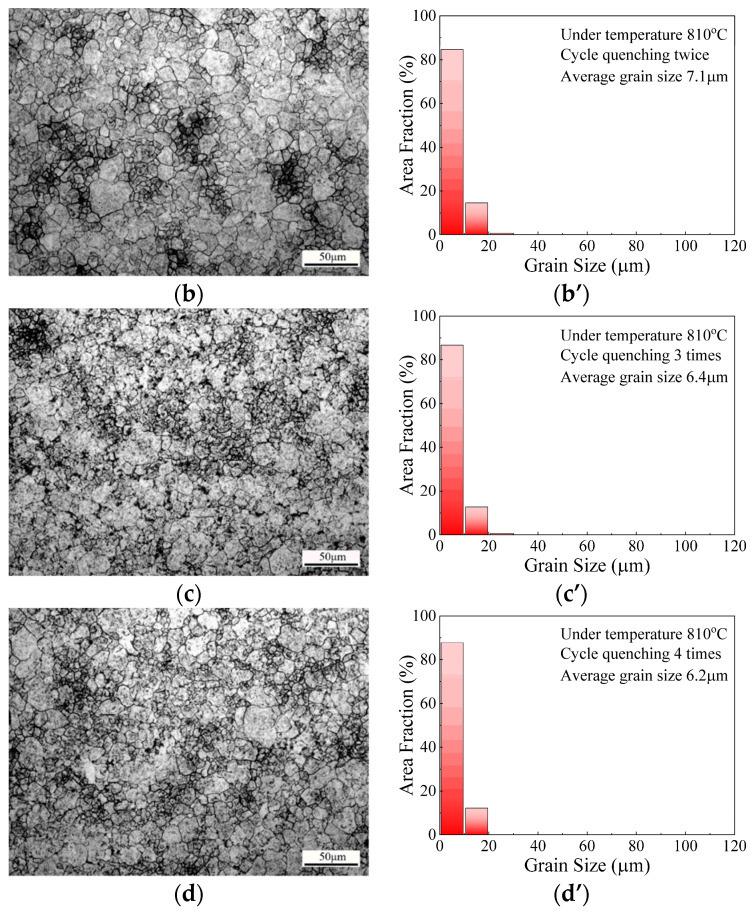
Microstructures and grain size distributions of specimens subjected to different cyclic quenching times at the optimal cyclic quenching temperature of 810 °C: (**a**,**a’**) once; (**b**,**b’**) twice; (**c**,**c’**) 3 times; (**d**,**d’**) 4 times.

**Figure 11:**
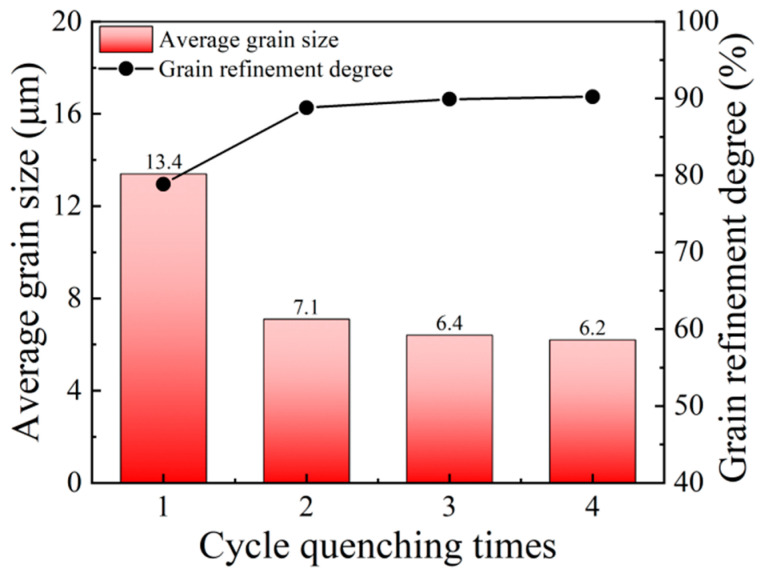
Variations in average grain size and grain refinement degree with cyclic quenching times.

Figure 10a–d illustrates the microstructures and grain size distributions of the specimens subjected to four different cyclic quenching times of one, two, three and four times at the fixed optimal cyclic quenching temperature of 810 °C with a fixed holding time of 5 min. It can be seen, from Figure 10, that, with the increase in cyclic quenching times, the austenite grains were refined and uniformly distributed in the microstructures. The average grain size at the cyclic quenching times were measured as 13.4 μm, 7.1 μm, 6.4 μm and 6.2 μm, respectively. The corresponding grain refinement degrees of the microstructures were calculated as 78.9%, 88.8%, 89.9% and 90.2%, respectively. From the variations in average grain size and grain refinement degree with cycle quenching times in Figure 11, with the increase in cyclic quenching times, the average grain size gradually decreased and the grain refinement degree increased. The reason underlying this trend is that, after the 65Mn low-alloy steel cyclic quenched once, the austenite grains were refined, which was accompanied by the breaking of martensite, as well as the increase in dislocations. The stored energy was higher in comparison with that of the microstructures after solid solution treatment and the driving force for recrystallization increased. With the increase in cyclic quenching times, the cyclic phase transformation between martensite and austenite occurred continuously in the microstructures, resulting in the increase in stored energy and driving force and further promoting grain refinement. Nevertheless, it is worthy to emphasize that, when the cyclic quenching times exceeded three times, the austenite grains were refined inconspicuously. Therefore, the average grain size decreased with the increase in cyclic quenching times, while the grain refinement degree first increased and then flattened out. The optimal cyclic quenching times for 65Mn low-alloy steel were determined to be four times.

## 5. Mechanical Performance Improvement through Refined Microstructures

### 5.1. Hardness

**Figure 12:**
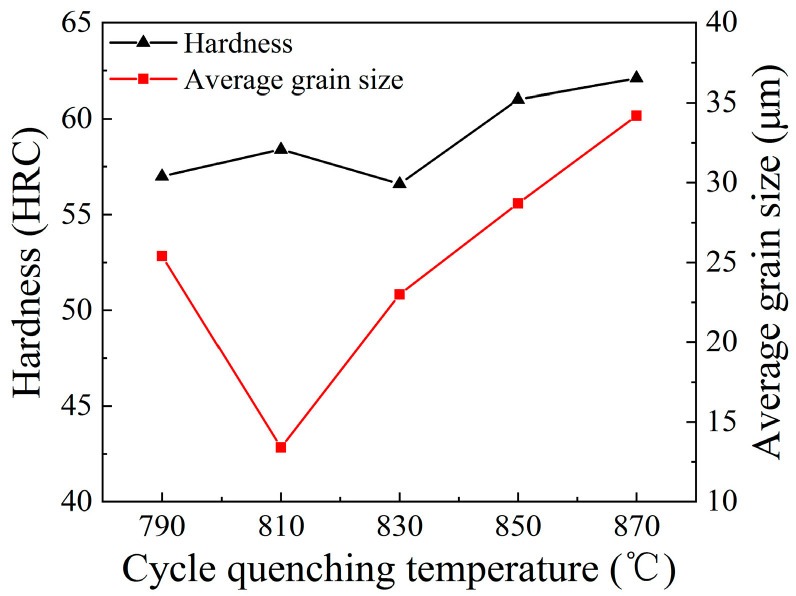
Relationship among hardness, average grain size and cyclic quenching temperature.

To analyze the effect of grain refinement on the hardness performance of 65Mn low-alloy steel, the hardness of the specimens subjected to cyclic heat treatment was characterized. Under the quenching temperatures of 790 °C, 810 °C, 830 °C, 850 °C and 870 °C, the hardness values of the specimens were measured as 57.0 HRC, 58.4 HRC, 56.6 HRC, 61.0 HRC and 62.1 HRC, respectively. Figure 12 exhibits the relationship among hardness, average grain size and cyclic quenching temperature. In Figure 12, we can see that a non-liner relationship between hardness and average grain size was observed. As the quenching temperature lowered below 830 °C, grain refinement strengthening was the primary reason for hardness improvement. However, when the quenching temperature exceeded 830 °C, with the increase in the temperature, more and more carbide particles, precipitated from the matrix during solid solution treatment, redissolved into austenite during the heating and holding process and the carbon content of austenite was raised. This led to the enrichment of carbon content in martensite after quenching. It is well known that the hardness improvement of martensite mainly results from the lattice distortion of martensite, with a higher carbon content making the distortion severer. Thus, the hardness was further improved under relatively a higher quenching temperature. This result also suggests that, once the quenching temperature of 65Mn low-alloy steel exceeded a certain threshold, the hardness was less affected by grain refinement, while it was mainly related to the quenching temperature. It can also explain why the hardness of the specimen after solid solution treatment was measured as 63.0 HRC, although the grain size was coarse. Therefore, the hardness of the specimens after cyclic phase transformation heat treatment was not only related to refined grain size but also associated with the quenching temperature.

**Figure 13:**
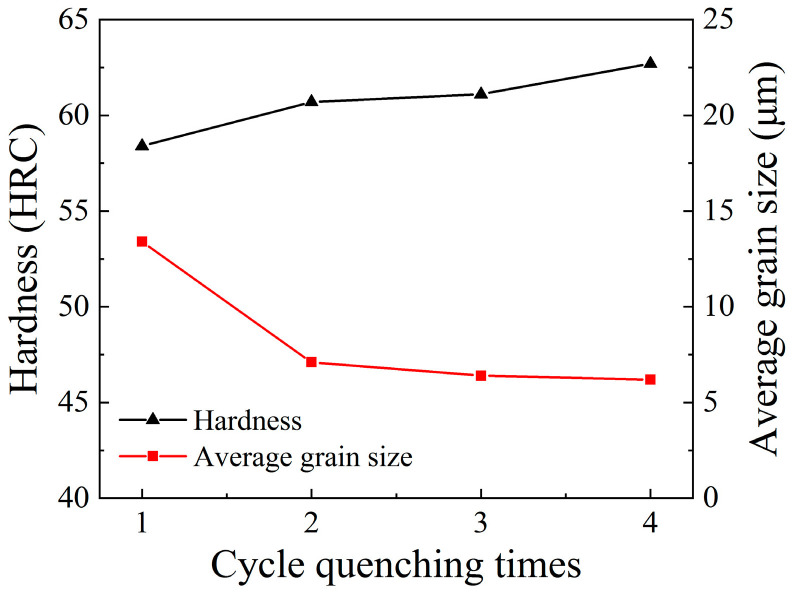
Relationship among hardness, average grain size and cyclic quenching times.

Similarly, under the cyclic quenching times of one, two, three and four times, the hardness values of the specimens were measured as 58.4 HRC, 60.7 HRC, 61.1 HRC, 62.7 HRC, respectively. The relationship among average grain size, hardness and cyclic quenching times is demonstrated in Figure 13. It can be seen, from Figure 13, that, with the increase in cycle quenching times, the hardness increased continuously with the decrease in average grain size. This is mainly due to the fact that, at the same optimal cyclic quenching temperature, grain refinement was prominently increased with the increase in cyclic quenching times.

### 5.2. Tensile Properties

**Figure 14:**
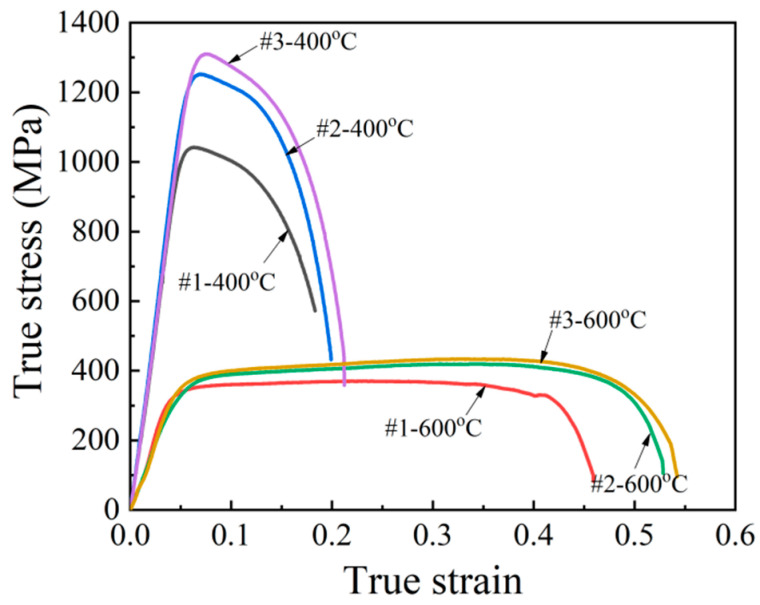
Tensile stress–strain curves of the tensile specimens at various temperatures with a fixed strain rate of 1 s^−1^.

**Figure 15:**
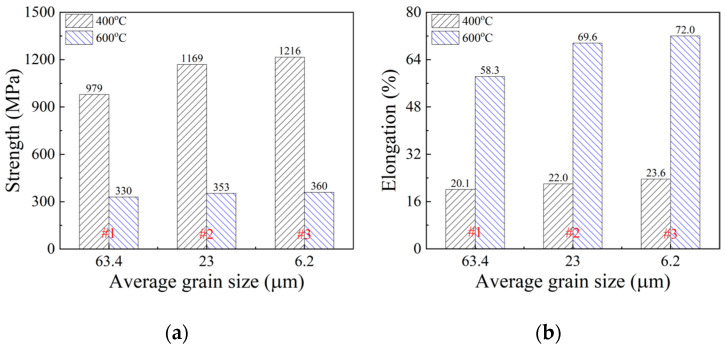
Relationships of tensile strength (**a**) and elongation (**b**) with average grain size.

The tensile properties of 65Mn low-alloy steel subjected to cyclic heat treatment under conditions #1, #2 and #3 were characterized, where #1 represents the tensile specimen subjected to solid solution treatment, #2 represents the specimen subjected to single quenching treatment under the temperature of 810 °C and #3 represents the specimen subjected to four time cyclic quenching treatment at the temperature of 810 °C. Figure 14 shows the obtained true stress–strain curves of the tensile specimens under the temperatures of 400 °C and 600 °C with a fixed strain rate of 1 s^−1^. It was found that these tensile specimens exhibited obvious plastic deformation behaviors. Meanwhile, owing to the significant difference in the refined microstructures, different heat treatment conditions had a strong influence on tensile properties. In order to better describe the tensile property difference under these heat treatment conditions, tensile strength and elongation were obtained from the stress–strain curves in Figure 14 and their relationships with average grain size are exhibited in Figure 15. It is noted that, at the temperatures of 400 °C and 600 °C, both the tensile strength and elongation increased with grain refinement. Comparing the tensile property of the specimen subjected to cyclic heat treatment with that of the specimen subjected to solid solution treatment, the tensile strength increased significantly from 979 MPa to 1216 MPa at 400 °C and the elongation also increased remarkably from 58.3% to 72% at 600 °C.

### 5.3. Wear Resistance

**Figure 16:**
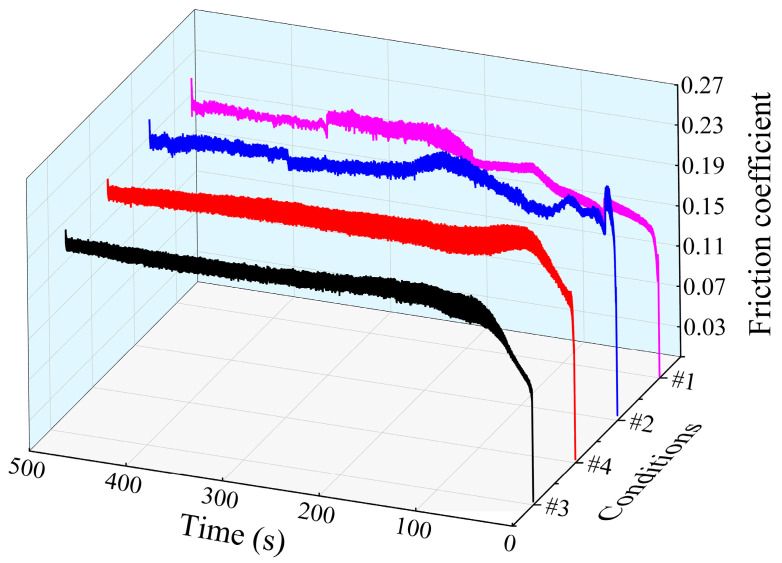
Variation in friction coefficient of different specimens with time.

The wear resistance of 65Mn low-alloy steel subjected to cyclic heat treatment under conditions #1, #2, #3 and #4 and were characterized, where conditions #1, #2 and #3 are the same as those mentioned in Section 5.2 and #4 represents the test specimen subjected to single quenching treatment under the cyclic quenching temperature of 830 °C. As an important indicator of wear-resistance performance, the friction coefficient varying with time of the test specimens subjected to heat treatment under conditions #1, #2, #3 and #4 are demonstrated in Figure 16. Generally, to a more stable friction coefficient corresponded better wear-resistance performance of the materials. A common feature in Figure 16 can be observed. All the friction coefficient variation in the studied specimens fluctuated greatly and gradually tended to be stable over time. This is mainly because, in the early running-in period, due to the unstable contact between the upper and lower testing rings, severe fluctuation in the speed of the lower testing ring appeared; further, as time passed, once the upper and lower testing rings made contact steadily, the friction coefficient also reached a stable value. In addition, by comparing the effect of various average grain sizes on the friction coefficient in Figure 16, it can be clearly seen that the fluctuation period of the friction coefficient decreased significantly with grain refinement in the running-in process and the amplitude in the stable period was also shortened. The results also indicate that the grain refinement microstructures achieved by cyclic phase transformation heat treatment could significantly improve the wear-resistance performance of 65Mn low-alloy steel.

**Figure 17:**
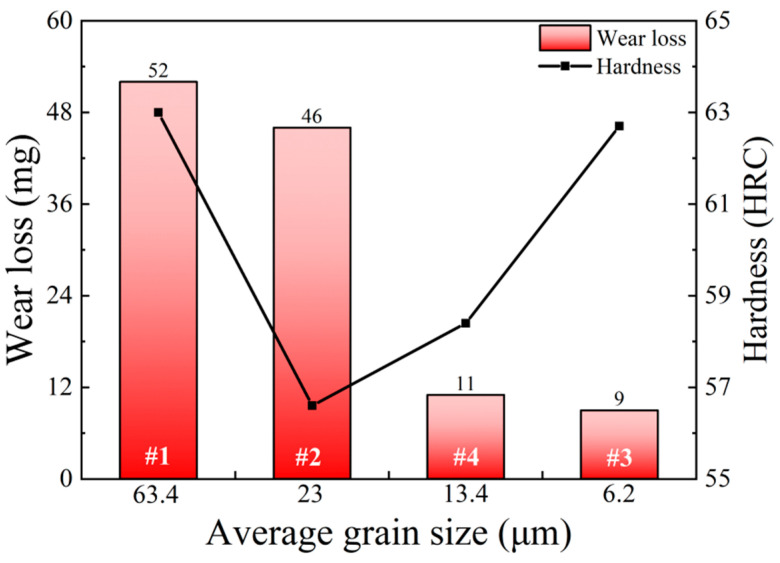
Relationships among wear loss, hardness and average grain size.

Additionally, the wear loss of the test specimens subjected to heat treatment under conditions #1, #2, #3 and #4 were also measured to characterize the wear resistance of 65Mn low-alloy steel. The wear-loss values corresponding to #1, #2, #4 and #3 were measured as 52 mg, 46 mg, 11 mg and 9 mg, respectively. Moreover, the relationship between wear loss and average grain size is depicted in Figure 17. It was found that wear loss decreased with grain refinement. Comparing the wear loss of the specimen subjected to cyclic phase transformation heat treatment with that of the specimen subjected to solid solution treatment, it can be clearly noted that wear loss decreased significantly from 52 mg to 9 mg. Such phenomenon indicates that the wear resistance of 65Mn low-alloy steel could be significantly enhanced by the refined microstructures obtained by cyclic phase transformation heat treatment. In addition, as can be seen from Figure 17, the relationship between wear loss and hardness is non-linear. According to reference [2], the wear process of 65Mn steel against Cu-based powder metallurgy material can be summarized by the following stages: the interface matching of asperities, the plastic deformation of the asperities and the fracture process of the asperities. For the test specimens subjected to heat treatment under conditions #1 and #2, both the values of wear loss and average grain size were relatively high, although the hardness of the specimen under condition #1 was much higher than that of the specimen under condition #2. This is because the fracture resistance of the specimens with coarse grain size was feeble and more asperities tended to break up in the wear process, thus resulting in the increase in wear loss. For the test specimens subjected to heat treatment under conditions #2, #4 and #3, with decrease in the average grain size, the wear-loss value decreased continually. This is attributable to the fact that, when the grain size was refined through cyclic heat treatment, the strength and ductility of specimens were improved synchronously. Correspondingly, the fracture resistance of the asperities was enhanced and wear resistance was also improved. It is worth noting that the wear loss of the specimens under conditions of #2, #4 and #3 decreased with the increase in hardness. This phenomenon can be explained by the fact that high hardness values could effectively restrain the plastic deformation of the asperities, thus avoiding the stress concentration and local crack propagation in the asperities. As a result, the wear resistance was further improved with the decrease in average grain size and increase in hardness. In short, the wear resistance of 65Mn low-alloy steel was affected by the synthesis reaction of grain refinement and hardness. Higher hardness and refined grain size contributed to the wear-resistance improvement of 65Mn low-alloy steel.

## 6. Conclusions

(1) The effects of cyclic quenching temperatures and quenching times on variations in average grain size were analyzed. With the increase in cyclic quenching temperature, the average grain size first decreased and then increased. With the increase in cyclic quenching times, the average grain size gradually decreased. The optimal cyclic quenching temperature and quenching times were determined to be 810 °C and four times.

(2) The relationships between hardness and average grain size varying with cyclic heat treatment parameters were revealed. With the increase in quenching temperature, hardness increased with grain refinement when the temperature was lower than 830 °C. Once the temperature exceeded 830 °C, hardness increased with the increase in temperature, owing to the enrichment of carbon content in martensite. With the increase in cyclic quenching times, hardness continuously increased with the strengthening of grain refinement.

(3) The tensile properties of 65Mn low-alloy steel subjected to various cyclic heat treatment conditions were characterized. The results indicate that grain refinement obtained from cyclic heat treatment could significantly improve the tensile strength and elongation simultaneously.

(4) The wear resistance of 65Mn low-alloy steel subjected to various cyclic heat treatment conditions were characterized by analyzing the friction coefficient and wear loss. It is shown that the fluctuation period and amplitude of friction coefficient decreased significantly with grain refinement. The relationships among wear loss, hardness and average grain size were revealed. It is shown that the wear resistance of 65Mn low-alloy steel was affected by the synthesis reaction of grain refinement and hardness and improved wear resistance was obtained with higher hardness and refined grain size.

## Figures and Tables

**Table 1:** Chemical composition (wt%) of 65Mn low-alloy steel.

C	Mn	Cr	P	S	Si	Ni
0.65	0.92	0.05	0.02	0.01	0.25	0.01

## Data Availability

Not applicable.
